# The research advances in Kirsten rat sarcoma viral oncogene homolog (KRAS)-related cancer during 2013 to 2022: a scientometric analysis

**DOI:** 10.3389/fonc.2024.1345737

**Published:** 2024-04-19

**Authors:** Yujie Huang, Daitian Zheng, Zhongming Zhou, Haiting Wang, Yanpo Li, Huihui Zheng, Jianhui Tan, Jingyao Wu, Qiuping Yang, Huiting Tian, Liuming Lin, Zhiyang Li, Tianyu Li

**Affiliations:** Department of Thyroid, Breast and Hernia Surgery, General Surgery, The Second Affiliated Hospital of Shantou University Medical College, Shantou, Guangdong, China

**Keywords:** *KRAS*, cancer, scientometrics, Bibliometrix, VOSviewer, Citespace

## Abstract

**Introduction:**

Cancer represents a significant global public health concern. In recent years, the incidence of cancer has been on the rise worldwide due to various factors, including diet, environment, and an aging population. Simultaneously, advancements in tumor molecular biology and genomics have led to a shift from systemic chemotherapy focused on disease sites and morphopathology towards precise targeted therapy for driver gene mutations. Therefore, we propose a comprehensive review aimed at exploring the research hotspots and directions in the field of *Kirsten rat sarcoma viral oncogene homolog* (*KRAS*)-mutant cancers over the past decade, providing valuable insights for cancer treatment strategies. Specifically, we aim to present an intellectual landscape using data obtained from the Web of Science (WoS) regarding *KRAS* mutation.

**Methods:**

Bibliometrix, VOSviewer, CiteSpace, and HistCite were employed to conduct scientometric analyses on national publications, influential authors, highly cited articles, frequent keywords, etc.

**Results:**

A total of 16,609 publications met the screening criteria and exhibited a consistent annual growth trend overall. Among 102 countries/regions, the United States occupied the vast majority share of the published volume. The journal *Oncotarget* had the highest circulation among all scientific publications. Moreover, the most seminal articles in this field primarily focus on biology and targeted therapies, with overcoming drug resistance being identified as a future research direction.

**Conclusion:**

The findings of the thematic analysis indicate that *KRAS* mutation in lung cancer, the prognosis following *B-Raf proto-oncogene, serine/threonine kinase* (*BRAF*) or *rat sarcoma* (*RAS*) mutations, and *anti-epidermal growth factor receptor* (*EGFR*)-related lung cancer are the significant hotspots in the given field. Considering the significant advancements made in direct targeting drugs like sotorasib, it is anticipated that interest in cancers associated with *KRAS* mutations will remain steadfast.

## 1 Introduction

GLOBOCAN 2020 estimates of cancer incidence and mortality suggest that cancer has been a growing burden on global health, and it is expected that the burden will have a 47% rise within 20 years (1). As one of the most frequently mutated oncogene *KRAS*, it is frequently seen in pancreatic, colorectal, lung adenocarcinomas, and urogenital cancers and is recognized as the founder carcinogenic mutation in the genome. *KRAS*, short for *Kirsten rat sarcoma viral oncogene homolog*, belongs to the *RAS* gene family, which also includes *HRAS* and *NRAS* (2). It is located on chromosome 12 and encodes a small GTPase protein that is involved in cell signalling pathways (3). *KRAS* plays a vital role in many cellular physiological processes by coupling membrane growth factor receptors with intracellular signalling pathways and transcription factors. *KRAS*, in combination with GTP, is switched on while GDP–bound is in an inactive state. Besides, the Guanine nucleotide exchange factor stimulates the transformation from the stable, inactive GDP-binding form to the active GTP binding form, while the conversion back to inactive form is mediated by the GTPase activating protein (4). In a pathological state, oncogene *KRAS* mutant locks *KRAS* in an active GTP binding state, which can activate a variety of signalling pathways, including RAF-MEK-ERK signalling pathway and PI3K-AKT-mTOR signalling pathway (5). Thus, it continuously produces proliferative and survival signalling through interacting with downstream effectors, thus, causing the development of cancers (6). The mutant subtypes of *KRAS* were identified as *KRAS^G12A^*, *KRAS^G12C^*, *KRAS^G12D^*, *KRAS^G12V^*, *KRAS^G12R^*, and *KRAS^G13D^* mutations or *KRAS* wild-type amplification, ect (7). Co-occurring genetic events, different *KRAS* mutation subtypes, and mutated *KRAS* allele content were the main drivers leading to direct clinical implications (8). In the past, *KRAS* had been viewed as “undruggable” due to the challenge of finding classical drug-binding pockets on the surface of *KRAS* proteins. At the same time, the presence of high concentrations of GTP in cells under physiological conditions and the high level of affinity between GTP and *KRAS* complicate the development of competitive inhibitors (9). Therefore, researchers turn to target *KRAS* indirectly, including its upstream and downstream signal effector, RNA interference, as well as synthetic lethal method, etc. Nevertheless, most strategies fail due to the lack of activity or selectivity (2, 5). In 2013, a crystallographic study revealed that the *KRAS^G12C^* contains a drug-capable pocket below the switch-II region, making it possible to design irreversible covalent inhibitors with higher potency, selectivity, and bioavailability (10). Fortunately, within ten years, decades of research finally yielded some clinical progress when the *KRAS^G12C^* inhibitor sotorasib was approved by the Food and Drug Administration in 2021 (11). Recently, adagrasib has been preliminarily proven to be efficient among patients with *KRAS^G12C^* mutated non-small cell lung cancer (NSCLC) in a phase II clinical trial (12). However, the emergence of drug resistance has always been a constant problem. For example, some drugs could only exert effects in a small subset of *KRAS* mutant cancers, and it is hard to balance between efficacy and toxicity. Besides, resistance in *KRAS* mutant targeting therapy and combination therapy is inevitable and tricky, including *KRAS* alternations and amplification, the influence from upstream or downstream regulators, aberrant cell cycle regulation, epithelial-to-mesenchymal transition (EMT), transition of pathological type, and so on (6, 9). Due to the great efforts in the exploration of *KRAS*, including expression, modification, mutations, and regulators, more and more researchers have come to realize its significance in the past decades.

Bibliometrix analysis could highlight regions of interest and remove unnecessary information, as well as help scholars quickly grasp the research hotspots and future directions (13, 14). In view of the promising prospects, a bibliometric analysis of the research of *KRAS*-mutant cancer should never have remained vacant. This publication aims to explore *KRAS*-related cancer research, enable researchers to understand the development history and extract the most essential information from the massive research.

## 2 Methods

### 2.1 Data collection

For this study, the data source is the WoS core collection, which is widely recognized as a reputable and comprehensive digital literature resource database, and it has garnered considerable acceptance among researchers, making it the preferred database for conducting bibliometric analyses (15). The subject terms “*KRAS*” and “cancer” were applied as central terms for related publications, and the searching strategies were as below: #1 TS=“*KRAS*” OR TS=“*KRAS2*” OR TS=“*Kirsten rat sarcoma*” OR TS=“*K$-RAS*” OR TS=“*Kirsten RAS*” OR TS=“*KRAS-4?*” OR TS=“*KRAS4?*” OR TS=“*KRAS4B*” #2 TS=“Tumor*” OR TS=“Neoplasm*” OR TS=“Neoplasia*” OR TS=“Cancer*” OR TS=“Malignant neoplasm*” OR TS=“Malignancy” OR TS=“Malignancies” OR TS=“Benign neoplasm*”. Certain restrictions were imposed on the data collection process, exclusively including articles or review articles published in the English language. Till March 26, 2023, a total number of 16,791 relevant publications were retrieved through the WoS Core Collection in the Science Citation Index Expanded (SCI-EXPANDED)—2003-present Edition. Moreover, 19 retracted publications and 63 publications that were published in 2023 were excluded for accurate analysis. A rigorous filtering process was applied to a vast pool of search results, resulting in the identification of 16,609 publications. Among these, 14,353 were articles, while 2,356 were review articles. The filtered literature will be downloaded in full record, including title, authors, source, abstract, keywords, etc. The filtering process is meticulously illustrated in **Supplementary Figure 1**, providing an in-depth visual representation of the steps involved.

### 2.2 Statistical analysis

Due to the research characteristics of numerous and fragmented, the concurrence of Bibliometrix makes out a delicate visualization to grasp the constantly changing academic (16). It plays a crucial role in integrating the research achievement during a specific time period and promoting the research progress (17). Importing the raw files into the Biblioshiny website, we obtained the preliminary information of these publications as delineated in **Supplementary Table 1**, including period, type of literature, number of literature, number of citations, number of authors, and the cooperation of authors and so on. Based on the above information, a preliminary judgment was made on whether the results met the inclusion criteria. After that, the bibliometric analysis, ranging from countries/regions, institutions, journals, authors, and keywords, was collected. VOSviewer is available for bibliometric mapping and presenting the connections between each other in a comprehensible way (18). Therefore, co-authorship analysis of countries and authors and co-occurrence analysis of keywords were conducted through VOSviewer (version 1.6.18). HistCite Pro is a software tool for drawing out a “Web of knowledge” (19). We applied HistCite (2.1) to obtain lists of the top ten publications ranked by local cited references (LCR) and local citation score (LCS). CiteSpace is a tool that embeds geographic visualization, time slice, and network clustering, which was developed by Chaomei Chen (20). Because of the quantitative restriction of CiteSpace (6.1.R2), it was mainly used to elicit the references and keywords with the most citation burst.

## 3 Results

### 3.1 Annual growth trend of publications

**Figure 1:**
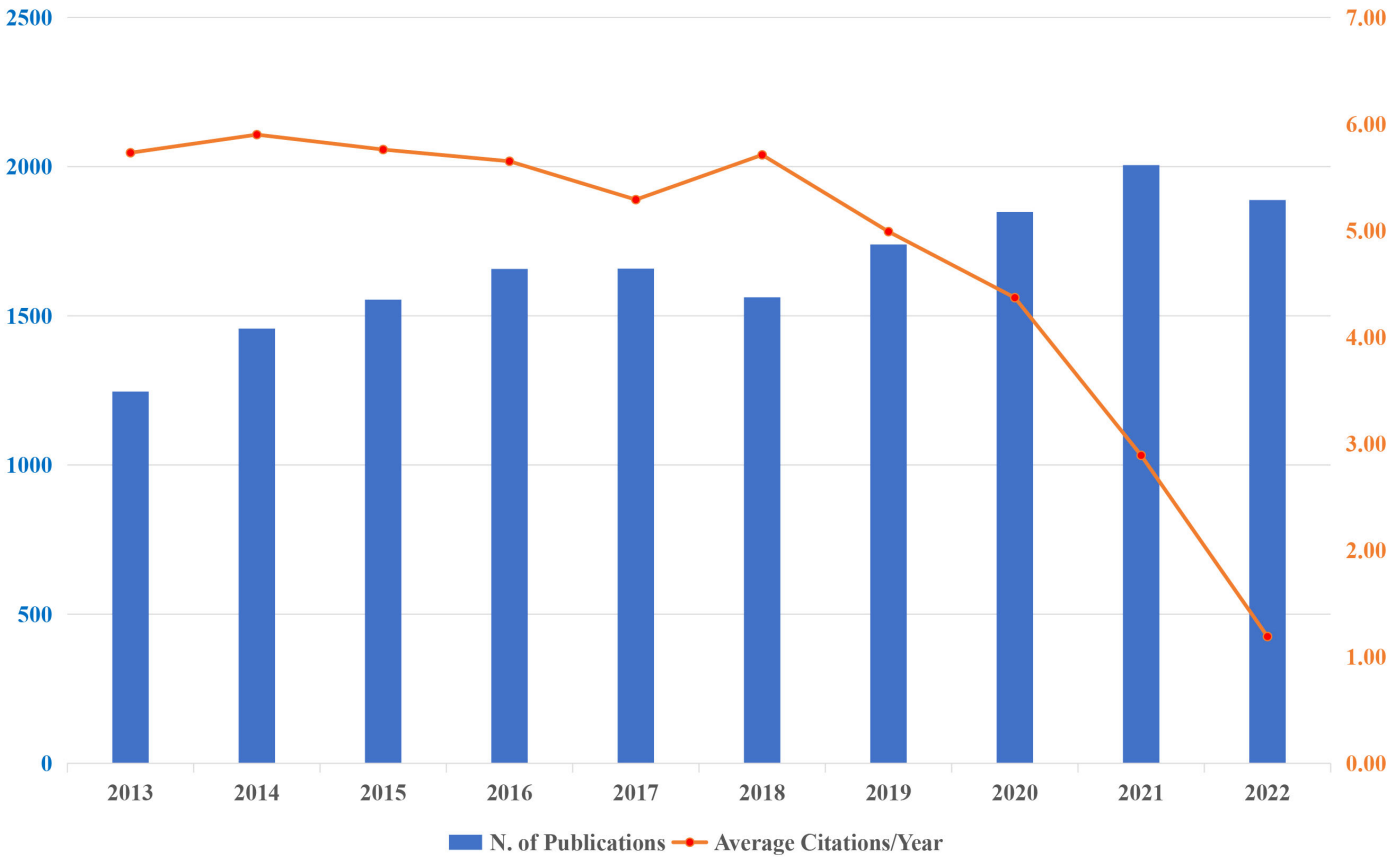
Annual publications output and corresponding average citations per year in the research of KRAS-related cancer during 2013 to 2022.

A total of 16,709 publications have been published across the past decade; the growth trend and corresponding mean citations are described in **Figure 1**. The blue bar signifies the annual scientific output, while the orange broken line illustrates the average citations per year. In broad terms, there has been a general trend of steady progress, albeit with a slight decline observed in 2018 and 2022. It displayed an upward trend from 1,246 documents (accounting for 7.46%) published in 2013 to 2,041 documents (accounting for 12.22%) published in 2021. Besides, 2021 (n=2005, accounting for 12.00%) was the year that contributed the most publication output. The median annual growth rate was 4.97%, reaching its peak in 2014 at 16.93% and hitting its lowest point in 2022 at -5.84%. Citation analysis involves analyzing citation patterns and metrics to assess the significance, visibility, and popularity of scientific works within a given field, as well as helps researchers identify influential publications, track research trends, measure the impact of specific researchers or institutions, and inform decisions regarding scholarly publishing, funding, and academic career advancement. Citation analysis involves analyzing citation patterns and metrics to evaluate the importance, visibility, and popularity of scientific works in a specific field. Looking at the broken line, it is not difficult to see that the citations are in proportion to citable years. The mean total citations per year, which exceeded 5.00 were from year 2013 to 2018. On average, the year 2014 had a maximum number of citations (citations=5.90), while 2022 had the lowest (citations=1.19).

### 3.2 Analysis of countries/regions and institutions

**Figure 2:**
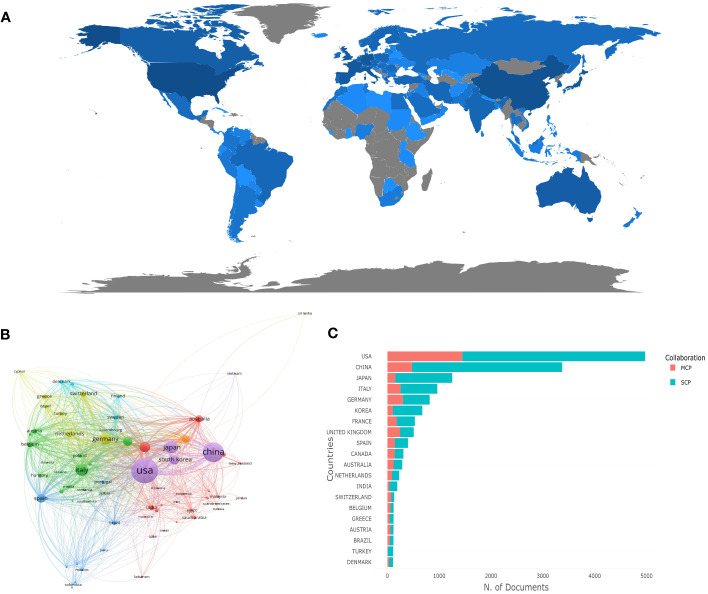
Visualization of countries/regions analysis in the research of KRAS-related cancer during 2013 to 2022. **(A)** Distribution of publications from different countries/regions. **(B)** The collaboration network between countries. **(C)** Top 10 countries’ publications partnerships.

Geographical distribution analysis highlights the involvement of various nations in a specific research field, shedding light on their contributions. In the case of cancer-associated *KRAS* research, the corresponding authors were distributed in 102 countries/regions. In **Figure 2A**, the software Bibliometrix was utilized to depict different countries’ production visually. The presence of blue color in countries or regions indicates the location of corresponding authors, while the intensity of the blue shade is directly proportional to the number of publications attributed to that particular country or region. A deeper shade of blue represents a higher number of published articles. Notably, the United States and China stand out as the regions with the most extensive representation, as indicated by their deep blue color. **Table 1** elicits the ten most prolific countries/regions and explains their cooperation in the research field, and the United States emerges as the forerunner in publications, with a substantial count of 4,972 (accounting for 29.76%). Following suit, China takes the following position with a commendable share of 20.18%, contributing 3,372 publications to the body of knowledge. Japan ranked third (n=1,251, accounting for 7.49%), followed by Italy (n=963, accounting for 5.76%) and Germany (n=816, accounting for 4.88%). However, the influence of a country’s population can not be ruled out when assessing research output. When it comes to the average article citations, the United States took into account both quantity and quality. On the other end of the spectrum, China presents the lowest average citations, with a value of 16.85, closely followed by Korea, with 17.80 citations. These findings underscore the diverse citation patterns across different regions, highlighting the significance of citation analysis to gauge the impact of scientific works. In **Figure 2B**, we employed VOSviewer, a powerful tool for visualizing collaboration networks, to map out the interconnectedness between countries effectively. Each node symbolizes a specific country, with the size of the node reflecting the volume of publications it has contributed. Additionally, the lines connecting the nodes represent the collaboration between countries, and the proximity of the lines indicates the level of cooperation. The closer the distance between the lines, the stronger the collaborative ties between the countries. To establish a threshold, set the minimum requirement for the number of documents from a specific country to five. After applying this criterion, a total of 70 documents successfully met the threshold when analyzed using VOSviewer. It is evident that these countries have engaged in close collaboration, resulting in the formation of seven distinct clusters. Particularly noteworthy is the United States, which demonstrates a strong level of collaboration with other countries, indicated by the highest total link strength of 4,921. **Figure 2C** distinguishes each country’s production from single-country publications (SCP) and multiple-country publications (MCP). To compare the collaborative competence between countries, we applied the ratio of MCP to SCP, as shown in the last column of **Table 1**. A higher ratio of MCP to SCP implies a greater degree of international collaboration in the research field. It suggests that researchers from different countries are actively collaborating and pooling their resources, knowledge, and expertise to produce scientific publications. Both the United Kingdom (MCP/SCP=0.477) and Canada (MCP/SCP=0.455) owed a ratio of more than 0.45, which shows that they have a relatively high cooperation level.

**Table 1:** Top 10 most productive countries/regions in the research of *KRAS*-related cancer during 2013 to 2022.

Rank	Countries/Regions	Publications	% of 16,709 publications	Total Citations	Average Article Citations	Total Link Strength	MCP/SCP
1	United States	4,972	29.76%	241,863	48.65	4,921	0.291
2	China	3,372	20.18%	56,812	16.85	1,587	0.141
3	Japan	1,251	7.49%	27,705	22.15	976	0.123
4	Italy	963	5.76%	29,163	30.28	1,852	0.265
5	Germany	816	4.88%	26,019	31.89	2,174	0.370
6	Korea	674	4.03%	11,998	17.80	596	0.154
7	France	528	3.16%	21,980	41.63	1,399	0.343
8	United Kingdom	509	3.05%	21,175	41.60	1,846	0.477
9	Spain	398	2.38%	12,568	31.58	1,483	0.359
10	Canada	308	1.84%	9,977	32.39	915	0.455

SCP, Single Country Publications; MCP, Multiple Country Publications.

In the analysis of institutions, **Table 2** provides a comprehensive list of the top ten most represented institutions, arranged in descending order according to their scientific output. At the top of the list was the University of Texas MD Anderson Cancer Center, with 1,650 publications, which accounted for 9.87% of the total 16,709 publications. Memorial Sloan Kettering Cancer Center is closely followed with an impressive number of 1,017 publications. In general, the top ten institutions for article production were predominantly from the United States and China.

**Table 2:** Top 10 most productive institutions in the research of *KRAS*-related cancer during 2013 to 2022.

Rank	Institutions	Country	Publications	% of 16,709 publications
1	The University of Texas MD Anderson Cancer Center	United States	1,650	9.87%
2	Memorial Sloan Kettering Cancer Center	United States	1,017	6.09%
3	Fudan University	China	757	4.53%
4	Dana-Farber Cancer Institute	United States	583	3.49%
5	Harvard Medical School	United States	549	3.29%
6	Johns Hopkins University	United States	534	3.20%
7	Sun Yat-sen University	China	532	3.18%
8	University of Michigan	United States	490	2.93%
9	University of California San Francisco	United States	485	2.90%
10	Shanghai Jiao Tong University	China	471	2.82%

### 3.3 Analysis of journals, publishers and research areas

Throughout the past ten years, 1,050 journals have been engaged on this subject altogether. **Table 3** presents a compilation of the top ten highly productive journals, accompanied by their respective h-index. The highest number of “h” such that an individual has published at least “h” papers was defined as the h-index, each of which has been cited at least “h” times. It has been widely applied across various disciplines and has proven to be a valuable tool for evaluating researchers, institutions, and even countries in terms of their research impact (21). *Oncotarget* (n=469) ranks first in publication volume, and *PLoS One* (n=447) and *Cancers* (n=438) were the other two journals that have an output of over 400. In view of the h-index, *Clinical Cancer Research* (h-index=87) stood out as the highest-quality journal on the list. To gain insight into the journals, Impact Factor and JIF Quartile were utilized for future comparison. The Law of Bradford, proposed by Samuel C. Bradford in 1934, describes the distribution of scientific literature across journals. It states that when articles are organized by subject relevance, they can be grouped into zones, with each zone containing a constant fraction of the total articles, which is of great help in identifying core journals in specific research fields (22). According to the Law of Bradford, Bibliometrix excavated a core journal list, as shown in **Supplementary Table 2**.

**Table 3:** Top 10 most productive journals in the research of *KRAS*-related cancer during 2013 to 2022.

Rank	Journals	Publications	% of 16,709 publications	H-index	IF/JIF Quartile (2022)
1	Oncotarget	468	2.80%	59	—
2	PLoS One	447	2.68%	53	3.752/Q2
3	Cancers	438	2.62%	32	6.575/Q1
4	Scientific Reports	310	1.86%	42	4.996/Q2
5	Clinical Cancer Research	308	1.84%	87	13.801/Q1
6	Frontiers in Oncology	287	1.72%	23	5.738/Q2
7	BMC Cancer	230	1.38%	32	4.638/Q2
8	Cancer Research	219	1.31%	57	13.312/Q1
9	Oncogene	212	1.27%	46	8.756/Q1
10	International Journal of Molecular Sciences	211	1.26%	31	6.208/Q1

IF, Impact Factor; JIF Quartile, Journal Impact Factor Quartile.

**Supplementary Figure 2** presents an analysis of publishers, specifically focusing on the top ten publishers sorted in descending order based on the number of publications. Springer Nature (n=2,906) and Elsevier (n=2,858) ranked top with a publication count that exceeded 2,800.

At the same time, the built-in function of WoS was used to have a list of the most active research areas in this knowledge domain. As shown in **Supplementary Table 3**, Oncology emerged as the most represented area with a significant volume of publications (n=7,962). Cell Biology (n=1,923) and Biochemistry Molecular Biology (n=1,547) ranked as the second and third most active research domains in this field of knowledge, respectively.

### 3.4 Analysis of authors

In terms of the authors’ analysis, a list of the most prolific authors were extracted from VOSviewer as shown in **Table 4**. Tabernero Josep from Spain occupied the highest volumn, who aimed at specific cancer proteins for the purpose of creating personalized treatments, cutting a striking figure in this area.

**Table 4:** Top 10 most productive authors in the research of *KRAS*-related cancer during 2013 to 2022. .

Rank	Authors	Publications	Total Citations
1	Tabernero Josep	59	131
2	Lenz Heinz-josef	49	2,249
3	Wang Jing	49	439
4	Nussinov Ruth	49	404
5	Zhang Wei	48	1,702
6	Wang Lei	48	1,324
7	Laurent-puig Pierre	48	539
8	Ladanyi Marc	48	416
9	Fassan Matteo	46	379
10	Kopetz Scott	46	298
10	Wistuba Ignacio i.	46	270

### 3.5 Analysis of articles and references

LCS represents the total number of citations received by a specific paper within a local dataset, which is a measure of the reference’s influence and impact within a narrower network. Similarly, global cited score (GCS) acrosses a broader spectrum beyond a specific dataset or local context. A high LCS for a piece of literature indicates its significance within the field and suggests that it may be considered a seminal article in a specific area of research. Therefore, a list of the articles with the most LCS was attained by HistCite and shown in **Table 5**. Douillard JY et al. testified that any patients could not benefit from panitumumab-FOLFOX4 treatment with activating *RAS* mutations, that is, expanding the scope of negative predictive biomarkers for *EGFR* therapy besides from *KRAS* mutations in exon 2 (23). In the same year, Ostrem JM et al. offered structure-based validation on the aspects of allosterically controlled GTP affinity and effector interactions of small molecules that could irreversibly bind to *KRAS* after G12C mutated (10). In 2014, Stephen AG et al. provided a comprehensive understanding of *RAS* biology and biochemistry (24). Heinemann V et al. proposed that FOLFIRI plus cetuximab may be more appropriate for patients with metastatic colorectal cancer with *KRAS* exon 2 wild-type through a phase III trial (25). Cox AD et al. discussed the possible directions for developing targeted drugs, such as blocking *RAS* membrane, downstream effector signalling, and utilizing synthetic lethal interactors (26). In 2017, Simanshu DK et al. sorted out the *RAS* effector, subcellular localization, spatial structure, and the function of *RAS* in human disease (4). Janes MR et al. described and identified a covalent G12C-specific inhibitor with high potency and selectivity for *KRAS^G12C^* (27). In 2019, Canon J et al. put forward that sotorasib could generate a pro-inflammatory tumor microenvironment and produce a lasting effect when combined with immune checkpoint inhibitors (28). Hong DS et al. conducted a phase I trial of sotorasib in *KRAS^G12C^*-mutant cancer patients and showed encouraging responses, which moved forward the subsequent Food and Drug Administration approval (29). Generally speaking, these articles show a tendency in drug development, drug efficacy, applicable population and so on.

**Table 5:** The top 10 articles with the most local citation scores in the research of *KRAS*-related cancer during 2013 to 2022.

Rank	Title	First Author, Year	Journal	LCS	GCS	LCS/GCS ratio (%)
1	Panitumumab-FOLFOX4 Treatment and RAS Mutations in Colorectal Cancer	Douillard JY, 2013	New England Journal of Medicine	806	1,622	49.69%
2	Drugging the undruggable RAS: Mission Possible?	Cox AD, 2014	Nature Reviews Drug Discovery	651	1,139	57.16%
3	K-Ras(G12C) inhibitors allosterically control GTP affinity and effector interactions	Ostrem JM, 2013	Nature	650	1,251	51.96%
4	The clinical KRAS(G12C) inhibitor AMG 510 drives anti-tumour immunity	Canon J, 2019	Nature	429	781	54.93%
5	FOLFIRI plus cetuximab versus FOLFIRI plus bevacizumab as first-line treatment for patients with metastatic colorectal cancer (FIRE-3): a randomised, open-label, phase 3 trial	Heinemann V, 2014	Lancet Oncology	394	1,236	31.87%
6	Genomic analyses identify molecular subtypes of pancreatic cancer	Bailey P, 2016	Nature	392	1,937	20.24%
7	Targeting KRAS Mutant Cancers with a Covalent G12C-Specific Inhibitor	Janes MR, 2018	Cell	372	606	61.39%
8	Dragging Ras Back in the Ring	Stephen AG, 2014	Cancer Cell	328	579	56.65%
9	KRAS (G12C) Inhibition with Sotorasib in Advanced Solid Tumors	Hong DS, 2020	New England Journal of Medicine	326	635	51.34%
10	RAS Proteins and Their Regulators in Human Disease	Simanshu DK, 2017	Cell	324	831	38.99%

LCS, local citations scores; GCS, global citations scores.

When it comes to LCR, it indicates the visibility and impact of that reference within a defined group of articles. An article with a higher LCR is more likely to be concerned about the field of *KRAS*-related cancer, which could find new trends from the article. In **Table 6**, a publication from *Cancers* written by Bontoux C et al. took the first lead in the LCR score. He published an update about the detection of *KRAS* mutations in NSCLC, including signalling pathways, epidemiology, prognosis, progress in drug, resistance mechanisms, molecular testing, rendering a broad perspective for the detection and treatment among *KRAS*-mutant NSCLC patients (30). Chen K et al. reviewed the *RAS*-mutant cancer. Then, they sorted out the target proteins, tumor model, and phase of the trial of different *RAS* inhibitors in detail, containing targeting upstream proteins, downstream proteins, and RNA interference (2). Huang LM et al. retrospected the potential mechanisms of resistance to *KRAS^G12C^* inhibitors and possible combination therapies, aiming to achieve precision treatment. Combination strategies to improve efficacy and delay resistance have been proposed, and combined with chemoradiotherapy, upstream molecules, downstream molecules, synthetic lethality screen, immune therapy, tumor metabolism therapy is advisable for less toxicity and better tolerance. He also rendered some resistance mechanisms, including the release of *ERK*-mediated feedback inhibition, EMT, the activation of bypasses, and subsequent *KRAS* mutations (5). Tang DL et al. also reviewed the strategies for overcoming primary or secondary resistance to *KRAS^G12C^*, including producing new *KRAS^G12C^* protein, activating wild-type *RAS*, inducing EMT, and inducing secondary genetic alterations. At the same time, some guidance on developing the next generation of drugs has been provided, such as identifying the off-target effects of G12C inhibitors to reduce nonspecific reactions, optimizing the vivo pharmacokinetic properties, obstructing different signalling pathways through drug combination, developing predictive biomarkers, etc. (31). Sveen A et al. derived implications from previous clinical trials and cohort studies that *KRAS* mutations may have a stronger effect on prognosis in patients with metastatic colorectal cancer, and discussed possible biomarkers to recognize resistance to *EGFR* inhibition (32). Kerk SA et al. summarized the metabolic networks regulated by mutations *KRAS* in colon, lung, and pancreatic tumors, focusing on the co-occurring mutations and the tumor microenvironment (33). Cekani E et al. sorted out the types and proportion of *KRAS* mutations according to different types and races of lung cancer first, and then the biological and co-mutations of *KRAS* in lung cancer were described, as well as the influence on prognosis (34). Uras IZ et al. provided the latest advances in anti-*KRAS* therapy for lung cancer, as well as mechanistic insights into biodiversity and potential clinical significance (8).

**Table 6:** Top 10 most locally cited references in the research of *KRAS*-related cancer during 2013 to 2022.

Rank	Title	First Authors, Year	Journal	LCR
1	Daily Practice Assessment of KRAS Status in NSCLC Patients: A New Challenge for the Thoracic Pathologist Is Right around the Corner	Bontoux C, 2022	Cancers	111
2	Emerging strategies to target RAS signaling in human cancer therapy	Chen K, 2021	Journal of Hematology & Oncology	110
3	KRAS mutation: from undruggable to druggable in cancer	Huang LM, 2021	Signal Transduction and Targeted Therapy	93
4	Oncogenic KRAS blockade therapy: renewed enthusiasm and persistent challenges	Tang DL, 2021	Molecular Cancer	89
5	KRAS: A Promising Therapeutic Target for Cancer Treatment	Wu HZ, 2019	Current Topics in Medicinal Chemistry	87
6	Biomarker-guided therapy for colorectal cancer: strength in complexity	Sveen A, 2020	Nature Reviews Clinical Oncology	85
7	Metabolic networks in mutant KRAS-driven tumours: tissue specificities and the microenvironment	Kerk SA, 2021	Nature Reviews Cancer	82
8	The molecular biology of pancreatic adenocarcinoma: translational challenges and clinical perspectives	Wang S, 2021	Signal Transduction and Targeted Therapy	80
9	Molecular Biology and Therapeutic Perspectives for K-Ras Mutant Non-Small Cell Lung Cancers	Cekani E, 2022	Cancers	80
10	TargetingKRASMutant Non-Small-Cell Lung Cancer: Past, Present and Future	Uras IZ, 2020	International Journal of Molecular Sciences	74

LCR, local cited references.

### 3.6 Analysis of keywords

**Figure 3:**
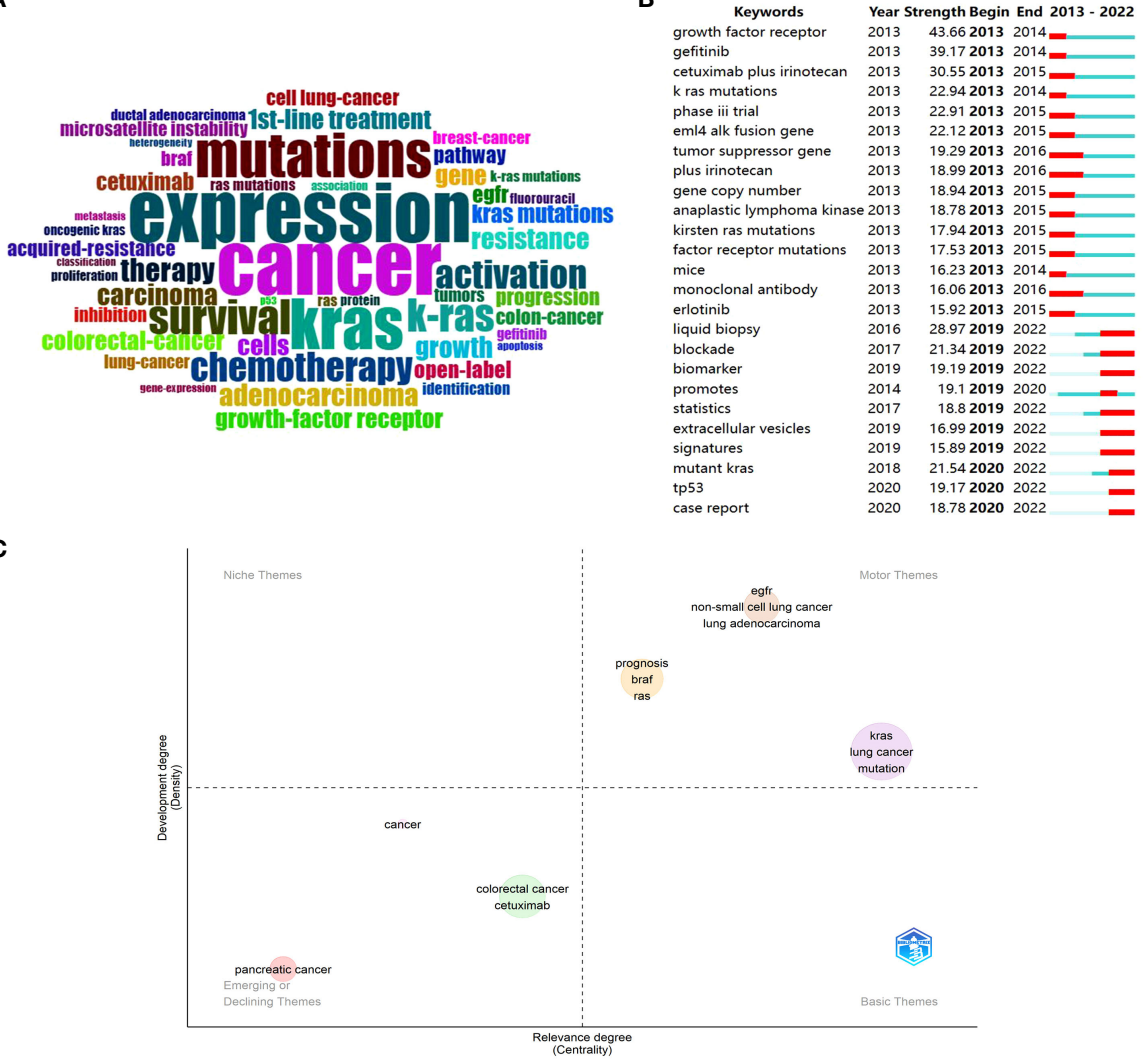
Visualization of keywords analysis in the research of KRAS-related cancer during 2013 to 2022. **(A)** Keyword cloud of the retrieved articles. **(B)** Top 25 keywords with the strongest citations bursts. **(C)** Thematic Map of authors’ keywords.

Keyword analysis is the process of identifying and analyzing the keywords that are relevant to a particular topic or subject. By conducting keyword analysis, researchers can gain insights into some important concepts, themes, or research areas within a given field. In **Figure 3A**, Bibliometrix depicted a word cloud based on the retrieved articles, and the font size increased in direct proportion to its frequency. Besides we also attached a tree map about the corresponding counts of keywords in **Supplementary Figure 3**. Except for the search terms “cancer”, “*kras*”, and “*k-ras*”, the top five most frequent words were “expression”, “mutations”, “survival”, “chemotherapy”, and “activation”, which pointed out that the focus in the research field may be on the expression and mutations of *KRAS* in cancer, and the subsequent effects on treatment and survival.

Through CiteSpace, a list of keywords with strong citation bursts was presented in **Figure 3B**, and the red bar denoted the actively cited period. It can be roughly divided into two periods: some keywords burst in the early stage, and the rest burst from 2019 until now. Sequenced by the total link strength, the top three burst words were “growth factor receptor”, “gefitinib”, and “cetuximab plus irinotecan” in the early stage. Combining with other keywords active at that time, the preliminary research concentrated on clinical trials and efficacy using drugs like gefitinib, cetuximab plus irinotecan, and erlotinib that target the *KRAS* upstream gene *EGFR* in cancer patients with *KRAS* mutations (35, 36). Currently, “liquid biopsy”, “mutant *KRAS*”, “blockade”, “biomarker”, “*tp53*”, “promotes”, etc. reflect that liquid biopsy has great potential for early cancer diagnosis, *KRAS* mutation detection and appropriate treatment strategies firstly (37, 38). Besides, the direct blockade therapy of *KRAS* gradually set off a research boom since the aggressive development of storasib and adagrasib (31).

In **Figure 3C**, the X-axis showed the relevance degree, whereas the development degree was indicated on the left Y-axis. It was divided into four quadrants, which were motor themes, niche themes, emerging or declining themes, and basic themes. Firstly, three clustered entities emerged within the motor themes, underscoring their vital significance in the research domain and yielding notable advancements. The biggest cluster is comprised of “*kras*”, “lung cancer”, and “mutation”, and the following one involves “prognosis”, “*braf*”, “*ras*”. The last one, located in the first quadrant, contains “*egfr*”, “non-small lung cancer”, “lung adenocarcinoma”. In other words, the investigation of *KRAS* mutation in lung cancer, prognosis evaluation for patients with *BRAF* or *RAS* mutations, and elucidation of *EGFR*’s role in NSCLC and lung adenocarcinoma represent pivotal research directions within this field that have made significant advancements. In the context of emerging or declining themes, the green cluster encompasses topics such as “colorectal cancer” and “cetuximab”, while the pink cluster is indicative of “pancreatic cancer”. Combining the searching results in WoS with the annual publication analysis, we can preliminary infer that the former is a declining theme, while the latter is an emerging theme.

**Figure 4:**
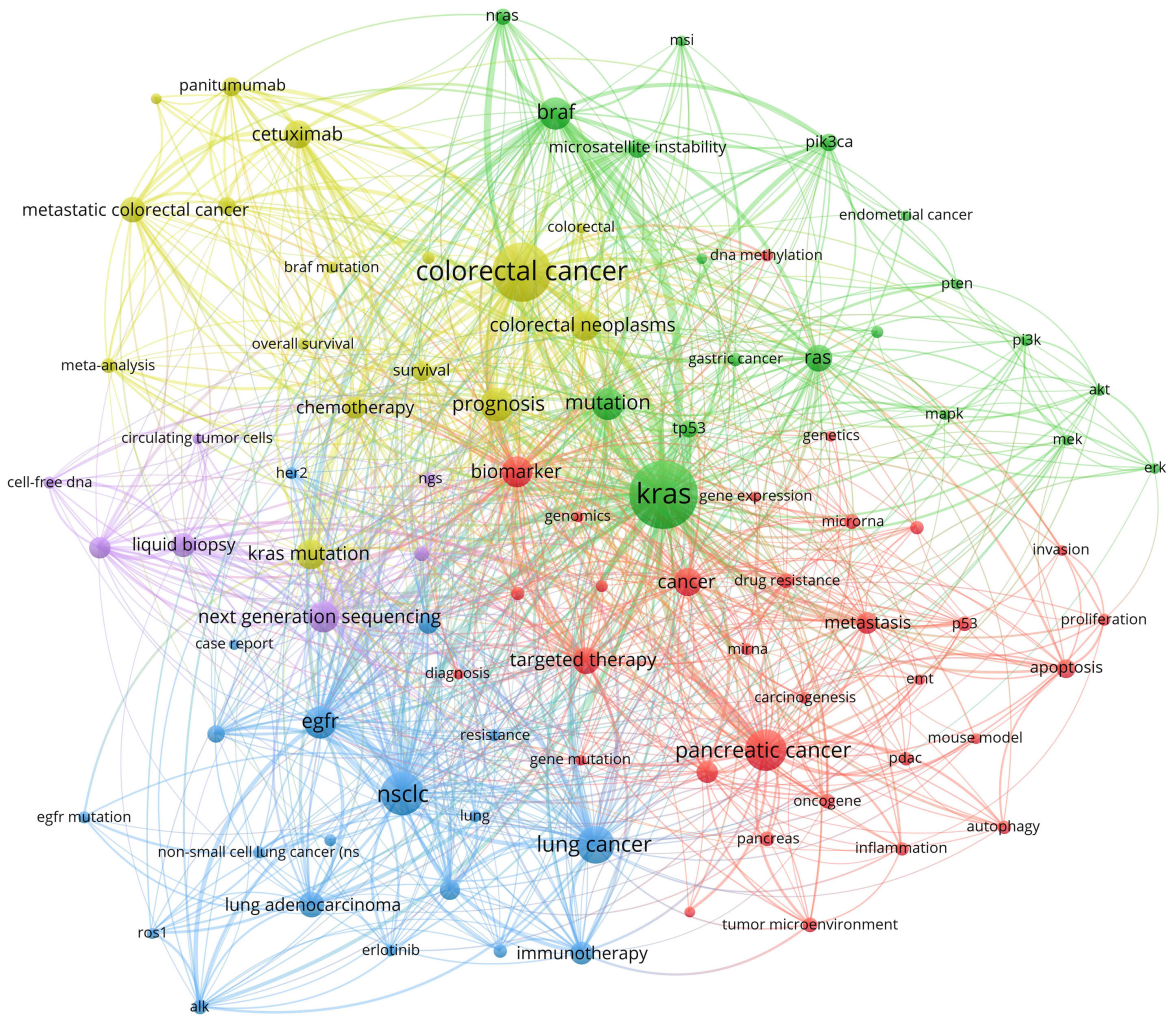
Co-occurrence analysis of keywords in the research of KRAS-related cancer during 2013 to 2022.

Apart from word frequency analysis, co-occurrence analysis was conducted to identify relationships and patterns between different terms based on their occurrence together in a corpus of documents. After setting the minimum number of occurrences of keywords at five using VOSviewer, we obtained a total of 18,222 keywords. For further analysis, we selected a subset of 93 keywords from this group with a minimum number of occurrences of a keyword at 60. The co-occurrence network was illustrated in **Figure 4**; the node size indicated the sum of occurrence times for each keyword. Furthermore, to enhance visual representation, the keywords were classified into five distinct clusters, with each cluster distinguished by a unique color. To have a better understanding of it, we sorted the keywords in descending order, as shown in **Table 7**. From cluster 1, it can be inferred that the focus likely pertained to gene expression and biological properties of *KRAS* in various cancer cells encompassing breast cancer, cholangiocarcinoma, and pancreatic cancer. This highlights the emphasis on molecular mechanisms underlying these cancers. Cluster 2 exhibited a significant enrichment of genes involved in the molecular pathway of *KRAS*, including *AKT*, *BRAF*, and *ERK*. This suggests a specific focus on gene interactions and pathways associated with *KRAS* across different cancers. Next, exploring the interplay between *EGFR* and *KRAS* and investigating how *EGFR*-combined *KRAS* mutations impact the efficacy and resistance of *EGFR* molecular inhibitors in lung adenocarcinoma was the primary goal of Cluster 3 (39, 40). Cluster 4 primarily focused on enhancing treatment strategies by evaluating the effectiveness of diverse therapies and combinations while identifying biomarkers capable of guiding personalized treatment approaches for patients with colorectal cancer. Lastly, cluster 5 concentrated on assessing the predictive value of *KRAS* and *BRAF* mutations within microsatellite-instability or microsatellite-stability subgroups in the context of colorectal cancer (41, 42). Additionally, attention was given to detecting the mutant status of *KRAS* using techniques such as liquid biopsy, next-generation sequencing, and circulating tumor DNA (37, 43, 44).

**Table 7:** Cluster of keywords in co-occurrence analysis in the research of *KRAS*-related cancer during 2013 to 2022.

Cluster	Color	Representative Keywords
1	▀	apoptosis, autophagy, biomarker, breast cancer, cancer, carcinogenesis, cholangiocarcinoma, diagnosis, dna methylation, drug resistance, emt, gemcitabine, gene expression, gene mutation, genetics, genomics, inflammation, invasion, metastasis, microrna, mirna, mouse model, oncogene, p53, pancreas, pancreatic cancer, pancreatic ductal adenocarcino, pdac, personalized medicine, proliferation, targeted therapy, tumor microenvironment
2	▀	akt, braf, endometrial cancer, erk, gastric cancer, heterogeneity, kras, mapk, mek, microsatellite instability, msi, mutation, nras, ovarian cancer, pi3k, pik3ca, pten, ras, tp53
3	▀	adenocarcinoma, alk, case report, egfr, egfr mutation, epidermal growth factor recept, erlotinib, her2, immunohistochemistry, immunotherapy, lung, lung adenocarcinoma, lung cancer, nsclc, pd-l1, resistance, ros1
4	▀	bevacizumab, braf mutation, cetuximab, chemotherapy, colorectal, colorectal cancer, colorectal neoplasms, irinotecan, kras mutation, meta-analysis, metastatic colorectal cancer, overall survival, panitumumab, prognosis, rectal cancer, survival
5	▀	cell-free dna, circulating tumor cells, circulating tumor dna, liquid biopsy, next generation sequencing, ngs, precision medicine

Red: Cluster 1; Green: Cluster 2; Blue: Cluster 3; Yellow: Cluster 4; Purple: Cluster 5.

## 4 Discussion

The passage conducted a scientometric analysis on a subset of 16,609 publications in correlation with *KRAS*-related cancer over the period from 2013 to 2022. For the past ten years, the publication volume in *KRAS*-related cancer has generally increased steadily, with the growth rate peaking in 2014 and the highest number of publications occurring in 2021. It is also observed that 2014 has the highest number of citations. The LCS analysis revealed that the articles published in 2013 and 2014 made up half of the list, which is consistent with the analysis of annual output. On one hand, extensive attention has been paid to the clinical trials on the efficacy among *KRAS*-mutant cancer patients, and findings indicated that *KRAS*-mutant patients could hardly benefit from anti-*EGFR* therapy like panitumumab plus FOLFOX4, highlighting the pressing need for other combination strategies and novel targeted medications (23). In the following year, phase III clinical trials brought great encouragement to the anti-*EGFR* therapy by suggesting that FOLFIRI plus cetuximab might be preferred for patients with metastatic colorectal cancer that is *KRAS* exon 2 wild-type (25). On the other hand, it should be noted that a drug-capable pocket in the *KRAS* switch-II region is a revolutionary discovery in the annals of research. Together with the possible strategies for developing targeted drugs, a research surge was elicited to investigate the development of targeted medications (10, 26).

In the countries/regions analysis, the United States held a good head in terms of both the publication volume and the average article citations. The University of Texas MD Anderson Cancer Center came in first place with a total of 1,650 publications among the most productive institutions, seven of which were from the United States and contributed 31.77% of the retrieved publications. It can be seen that the United States has a strong strength in the field of KRAS mutation-related cancer research and has achieved remarkable results. Besides, China stood out among developing nations in the study of *KRAS*-mutant cancer, for it was the only developing nation to be included on the list. However, there are relatively big structural problems in the quantity and output proportion of top-quality papers in China on account of its lowest citations. However, the good news is that Wang L, who has an h-index of 36, has entered the list of the most influential authors and the sole Chinese author. Within the collaboration network, the United States sharply exceeds other countries/regions with a total link strength of nearly 5,000. This outcome demonstrates the value of international cooperation from the standpoint that close-country cooperation is one of the factors contributing to its most significant output.

The journal analysis could evaluate and analyze the performance of journals within a specific field of study and help researchers make informed decisions about journal selection, publishing strategies, and research evaluation. For the analysis of the most prolific journal, *Oncotarget*, *PLoS One*, *Cancers* were the big three and contributed more than 2.50% of the total publications. Moreover, the journal *Clinical Cancer Research* ranked first in IF and h-index in *KRAS*-related cancer research. Additionally, we used the Law of Bradford to compile a list of the major forces contributing to this field, shown in **Supplementary Table 2**.

For articles and references analysis, the most locally cited articles could be divided into three categories: i) Clarify the biological characteristics and interplay effectors of *KRAS*: Stephen AG, Cox AD, and Simanshu DK et al. provided in-depth insights into the functional mechanisms, Interaction effector, and post-translational modification. What is more, the following was a thorough review and discussion on the current progress of *KRAS* targeted approaches, including restoring GTP hydrolysis, altering *RAS* localization, targeting *RAS* post-translational modification, targeting upstream/downstream effectors, directly attacking *RAS* proteins, and utilizing synthetic lethal screening, thus, offering a theoretical underpinning and possible direction for the development of *KRAS*-targeted medications (4, 24, 26). ii) Develop *KRAS*-targeted drugs: Ostrem JM et al. reported a new allosteric pocket of *KRAS* protein called S-IIP, which could put more weight on the preference towards GDP and lead to cumulative of inactive *KRAS* (10). Fortunately, after five years, Janes MR et al. designed and identified a covalent G12C-specific inhibitor based on the valent G12C-specific inhibitor S-IIP, and it has shown great potential to induce tumor regression *in vivo* (27). iii) Conduct clinical trials on *KRAS*-mutant patients: FOLFIRI plus cetuximab may be the first-line regimen for patients with *KRAS* exon 2 wild-type colorectal cancer, according to a phase III clinical trial (25). Canon J et al. demonstrated the anti-tumor activity of *KRAS^G12C^* inhibitor sotorasib in the first dosing cohorts and represents a potentially transformative therapy for patients for whom effective treatments are lacking (28). Hong DS et al. conducted a phase I clinical trial of sotorasib among patients with advanced solid tumors carrying the *KRAS^G12C^* mutation (29). Moreover, the first concern that stands out in the subset of retrieved literature is to overcome drug resistance, and the second concern is to explore *KRAS* lung cancer. In light of the resistance, Huang LM et al. and Tang DL et al. put forth several key mechanisms elucidating the resistance observed in the context of the mutated *KRAS^G12C^* protein upon treatment with ARS-1620. Firstly, the presence of this mutated protein leads to the adaptation of cancer cells, allowing them to tolerate the effects of ARS-1620. Secondly, the activation of multiple receptor tyrosine kinases (*RTKs*) triggers a feedback loop reactivating the wild *RAS* pathway, thereby conferring resistance to both ARS-1620 and sotorasib. Thirdly, the induction of EMT plays a role in the development of resistance to sotorasib or ARS-1620, principally through the activation of the *PI3K* pathway. Lastly, secondary genetic alterations significantly increase the likelihood of acquiring resistance to these targeted therapies (5, 31). When it comes to *KRAS*-mutant lung cancer, researchers elucidated the importance of accurate and timely determination of *KRAS* status in guiding treatment decisions for NSCLC. Novel targeted therapies for *KRAS*, including small molecule inhibitors and immunotherapy, may improve the prognosis of NSCLC patients. Besides, the importance of combination therapy, such as simultaneous inhibition of downstream effects and strategies based on synthetic lethal properties to overcome the inherent resistance to *KRAS*-targeted therapy, has been emphasized (8, 30, 34).

Keyword analysis helps identify emerging research areas, track the evolution of scientific topics, and facilitate literature reviews. Burst keywords show that previous studies have concentrated on assessing the effectiveness of *EGFR* inhibitors and investigating combination therapies among patients with *KRAS*-mutant cancers. In recent years, the hotspots of research have shifted to the means of early screening for *KRAS* mutations and the *KRAS*-mutant effect on prognosis. For example, *KRAS* mutation predicts poor prognosis in rectal cancer patients undergoing neoadjuvant chemoradiotherapy (45). These findings highlight the importance of KRAS mutation as a prognostic factor and provide a theoretical basis for the development of targeted therapeutic strategies against KRAS mutation. Thematic map analysis has uncovered crucial research directions that have made notable progress within the field, including the investigation of *KRAS* mutation in lung cancer, the assessment of prognosis for *BRAF* or *RAS* mutations, and the clarification of *EGFR*’s involvement in NSCLC and lung adenocarcinoma. Furthermore, it is imperative to draw attention to the emerging theme of *KRAS*-related pancreatic cancer. Sticking to *KRAS*-related pancreatic cancer research not only contributes to a deeper understanding of the pathobiology of pancreatic cancer but can also promote the development of treatment strategies, the refinement of individualized therapy, and the advancement of translational medicine. Finally, co-occurrence analysis is of essential help in obtaining cumulative knowledge of a research sub-population. In each of the five clusters, the primary hotspots are sorting out the biological properties, studying the molecular pathways, exploring the interaction between *EGFR* and *KRAS*, developing therapeutic strategies for colorectal cancer, and applying techniques to detect *KRAS* mutational status, respectively.

Given the formidable challenge posed by *KRAS*-mutant cancers, it is imperative to provide a concise overview of drug classifications, resistance mechanisms, and potential strategies to overcome them. Therapeutic drugs for *KRAS* mutations can be categorized into various types, such as direct or indirect targeting of *KRAS* inhibitors, immunotherapy, *KRAS* degraders and toxins, small interfering RNAs, etc. (9, 11, 46). Despite the availability of treatment options, cancer cells display remarkable adaptive capabilities by adjusting their states to develop resistance. There are three main mechanisms of resistance to *KRAS*-targeted therapy: i) Acquired *KRAS* mutations, which affect the binding sites of *KRAS* inhibitors, such as sotorasib and adagasib, particularly impacting amino acid residues at positions 12, 68, 95, and 96 of *KRAS^G12C^* (11). Additionally, long-term exposure to sotorasib or adagasib can lead to the development of 12 different secondary *KRAS* mutations, resulting in the production of new *KRAS^G12C^* in response to suppressed mitogen-activated protein kinase output (47). ii) Feedback activation of *KRAS* upstream and downstream signaling pathways, also known as adaptive resistance, can diminish the effect of *KRAS* inhibition by activating upstream or downstream mediators and other negative regulatory factors, leading to treatment ineffectiveness, recurrence, and progression. iii) EMT and adenocarcinoma transformation are potential mechanisms of acquired resistance that increase the mobility and invasiveness of tumor cells. Furthermore, tumor cells can evade targeted therapies by altering their pathological morphology to lose dependency on the original oncogenic driver. Besides, cancer cells can also promote tumor development by regulating cell cycle signaling and immune mechanisms (9). Due to several reasons such as gene mutations, drug metabolism, changes in drug targets, enhanced drug efflux pathways, and recombination of cell signaling pathways, monotherapy over a long period can easily lead to drug resistance. Therefore, rational and dynamic combination strategies are necessary to maintain long-lasting anti-tumor effects. Combination therapy is an important approach to overcome drug resistance due to its synergistic effects, delay in resistance development, and reduction of side effects. For instance, simultaneous inhibition of *EGFR* and *KRAS^G12C^* is necessary in colorectal cancer to overcome resistance to *KRAS^G12C^* blockade (48). Additionally, combining Src homology phosphatase 2 inhibitors with *KRAS^G12C^* inhibitors shows promise. Furthermore, combining sotorasib with anti-PD-1 therapy can promote T-cell activation and long-term tumor-specific immune responses (9, 11). Potential strategies for combination therapy include combining Tyrosine Kinase Inhibitors, Immune Checkpoint Inhibitor, rapamycin mTOR inhibitors, CDK4/6 inhibitors, Janus kinase (JAK) inhibitors, among others (46). For example, in *KRAS*-mutated lung adenocarcinoma cells, the secretion of proinflammatory cytokines, including interleukin-6, activates JAK1 and JAK2 through glycoprotein 130 in an autocrine loop, thus, contributing to malignant progression. Moreover, JAK1 inhibition has been shown to reduce the progression of *KRAS* mutant adenocarcinoma in mice (49). Further research is needed to determine the most effective combination strategy for patients. Developing biomarkers for personalized and appropriate treatments poses a new challenge (50). Some researchers believe that utilizing deep learning algorithms with artificial intelligence to integrate clinical data and predict resistance pathways is one pathway towards achieving personalized medicine and improving decision-making (51).

Based on the evidence reviewed above, the future direction of the work could be seeking early monitoring of *KRAS* mutations, elucidating the prognostic value of *KRAS* mutation, and conducting clinical trials for better combination strategies. Additionally, more research is required to develop targeted medications and combat drug resistance. Last but not least, we need to address the fact that *KRAS*-mutant NSCLC has garnered significant attention and interest among researchers.

## 5 Conclusion

The *KRAS* gene has undergone extensive investigation for several decades, with its pivotal involvement in the pathogenesis and progression of various cancers presenting substantial avenues for research. Unavoidably, the article has flaws that need to be addressed. First, WoS covered a wide range of publications from different fields and was accepted by researchers, so we chose WoS as the retrieval database but neglected other databases like EMBASE, PubMed, SCOPUS, and so on (15, 52). Besides, the exclusion of certain non-index sources contributes to a relatively lower searchability and impact in academic research. Therefore, neglecting those non-index sources may not make the research more comprehensive and diverse. Secondly, during the retrieval process, non-English publications were excluded for the sake of subsequent analysis convenience, potentially resulting in the exclusion of articles published in languages other than English, such as French, German, Spanish, etc. This exclusion may introduce a certain degree of bias in the subsequent country analysis, author analysis, and other analyses. Moreover, owing to the inherent publication bias observed in medical literature, which is often influenced by public interest (53), and displays a tendency to favor articles with positive outcomes (54), additional factors such as institutional prestige or author reputation are likely to exert an influence as well (55). These factors collectively contribute to shaping the composition of the retrieved document collection. Significantly, it is crucial to acknowledge that relying solely on citation as a measure of influence can undermine the credibility of the results. Generally speaking, earlier published articles receive more citations than later ones. In addition, research suggests that publications with a clinical research focus receive significantly fewer citations than those with a basic science subject (56). Beyond that, open-access articles usually acquire higher citations than traditionally accessed articles (57). An even more delicate question is that the outcome of literature analysis may also vary slightly from different software due to version issues. These can be achieved through the inclusion of non-English literature, incorporation of diverse information sources, and meticulous scrutiny and rectification of potential biases, or by making some appropriated interpretation and discussion.

In this study, a scientometric analysis was executed on articles delineating *KRAS*-associated cancer spanning the years 2013 to 2022, systematically exploring temporal trends in annual publication growth, geographical distribution of research endeavors, affiliations of contributing institutions, publishing journals, contributing authors, seminal articles, and keyword analysis. The amalgamation of literature and keyword analyses has unveiled salient themes within this domain of investigation, prominently encompassing the surveillance of *KRAS* mutations, the development of targeted therapeutic modalities, and the surmounting of mechanisms conferring resistance to pharmacological interventions. Consequently, this research endeavor elucidates the landscape of *KRAS*-mutant cancers, serving as a catalyst for intensified exploration by the scientific community, particularly in light of the escalating incidence of cancer.

## Data availability statement

The original contributions presented in the study are included in the article/**Supplementary Material**. Further inquiries can be directed to the corresponding authors.

## Author contributions

YH: Writing – original draft, Investigation, Conceptualization. DZ: Writing – review & editing, Formal Analysis. ZZ: Writing – review & editing, Formal Analysis. HW: Writing – review & editing, Validation. YL: Writing – review & editing, Validation. HZ: Writing – review & editing, Validation. JT: Writing – review & editing, Validation. JW: Writing – review & editing, Validation. QY: Writing – review & editing, Validation. HT: Writing – review & editing, Software. LL: Writing – review & editing, Software. ZL: Writing – review & editing, Supervision. TL: Writing – review & editing, Supervision.
