# Supplementary material for: The research advances in Kirsten rat sarcoma viral oncogene homolog (KRAS)-related cancer during 2013 to 2022: a scientometric analysis
Source: Front Oncol. 2024 Apr 19;14:1345737. doi: 10.3389/fonc.2024.1345737 (PMC11066287; doi:10.3389/fonc.2024.1345737)
Supplement: Supplementary file 1 [file DataSheet_1.docx]

Supplementary Material

**Supplementary Table 1** | Main information of the research in *KRAS*-related cancer during 2013 to 2022.

| Category | Specific Standard Requirements |
| --- | --- |
| Research database | Web of Science core collection |
| Citation indexes | SCIE |
| Searching period | January 2013 to December 2022 |
| Document types | “Article” or “Review Article” |
| Language | “English” |
| Data extraction | Export with full record and cited references in plain text format |
| Sample size | 16,709 |
| Annual growth rate % | 4.73 |
| Articles average age | 5.16 |
| Average citations per article | 31.88 |
| References | 331,462 |
| Keyword plus | 16,308 |
| Author’s keywords | 18,232 |
| Authors | 76,363 |
| Authors of single-authored articles | 156 |
| Single-authored documents | 179 |
| Co-authors per article | 9.96 |
| International co-authorship % | 27.05 |

**Supplementary Table 2 |** The core journals in the research of *KRAS*-related cancer during 2013 to 2022 according to the Law of Bradford.

| Rank | Journals | Publications | H-index | Total Citations |
| --- | --- | --- | --- | --- |
| 1 | Oncotarget | 468 | 59 | 14,199 |
| 2 | Plos One | 447 | 53 | 11,716 |
| 3 | Cancers | 438 | 32 | 4,984 |
| 4 | Scientific Reports | 310 | 42 | 6,510 |
| 5 | Clinical Cancer Research | 308 | 87 | 23,974 |
| 6 | Frontiers in Oncology | 287 | 23 | 2,317 |
| 7 | BMC Cancer | 230 | 32 | 4,159 |
| 8 | Cancer Research | 219 | 57 | 11,641 |
| 9 | Oncogene | 212 | 46 | 6,786 |
| 10 | International Journal of Molecular Sciences | 211 | 31 | 3,857 |
| 11 | Lung Cancer | 191 | 41 | 5,463 |
| 12 | Nature Communications | 179 | 54 | 9,170 |
| 13 | Oncology Letters | 176 | 20 | 1,801 |
| 14 | British Journal of Cancer | 159 | 46 | 6,700 |
| 15 | International Journal of Cancer | 151 | 40 | 4,727 |
| 16 | Journal of Thoracic Oncology | 138 | 52 | 7,092 |
| 17 | Annals of Oncology | 136 | 67 | 14,157 |
| 18 | Proceeding of the National Academy of Sciences of the United States of America | 125 | 51 | 7,920 |
| 19 | Anticancer Research | 121 | 24 | 1,858 |
| 20 | Molecular Cancer Therapeutics | 120 | 33 | 3,304 |
| 21 | Modern Pathology | 112 | 37 | 4,381 |
| 22 | World Journal of Gastroenterology | 112 | 38 | 4,422 |
| 23 | Gastroenterology | 111 | 55 | 8,483 |
| 24 | Human Pathology | 110 | 26 | 2,090 |
| 25 | Cancer Letters | 106 | 35 | 3,762 |
| 26 | Cancer Discovery | 105 | 63 | 13,297 |
| 27 | European Journal of Cancer | 103 | 37 | 4,268 |
| 28 | Cancer Medicine | 101 | 23 | 1,499 |
| 29 | Oncotargets and Therapy | 98 | 21 | 1,459 |

**Supplementary Table 3** | The top 5 most productive research fields in the research of *KRAS*-related cancer during 2013 to 2022.

| Rank | Research Field | Publications | % of 16,709 publications |
| --- | --- | --- | --- |
| 1 | Oncology | 7,962 | 47.65% |
| 2 | Cell Biology | 1,923 | 11.51% |
| 3 | Biochemistry Molecular Biology | 1,547 | 9.26% |
| 4 | Pathology | 1,463 | 8.76% |
| 5 | Multidisciplinary Sciences | 1,226 | 7.34% |

**
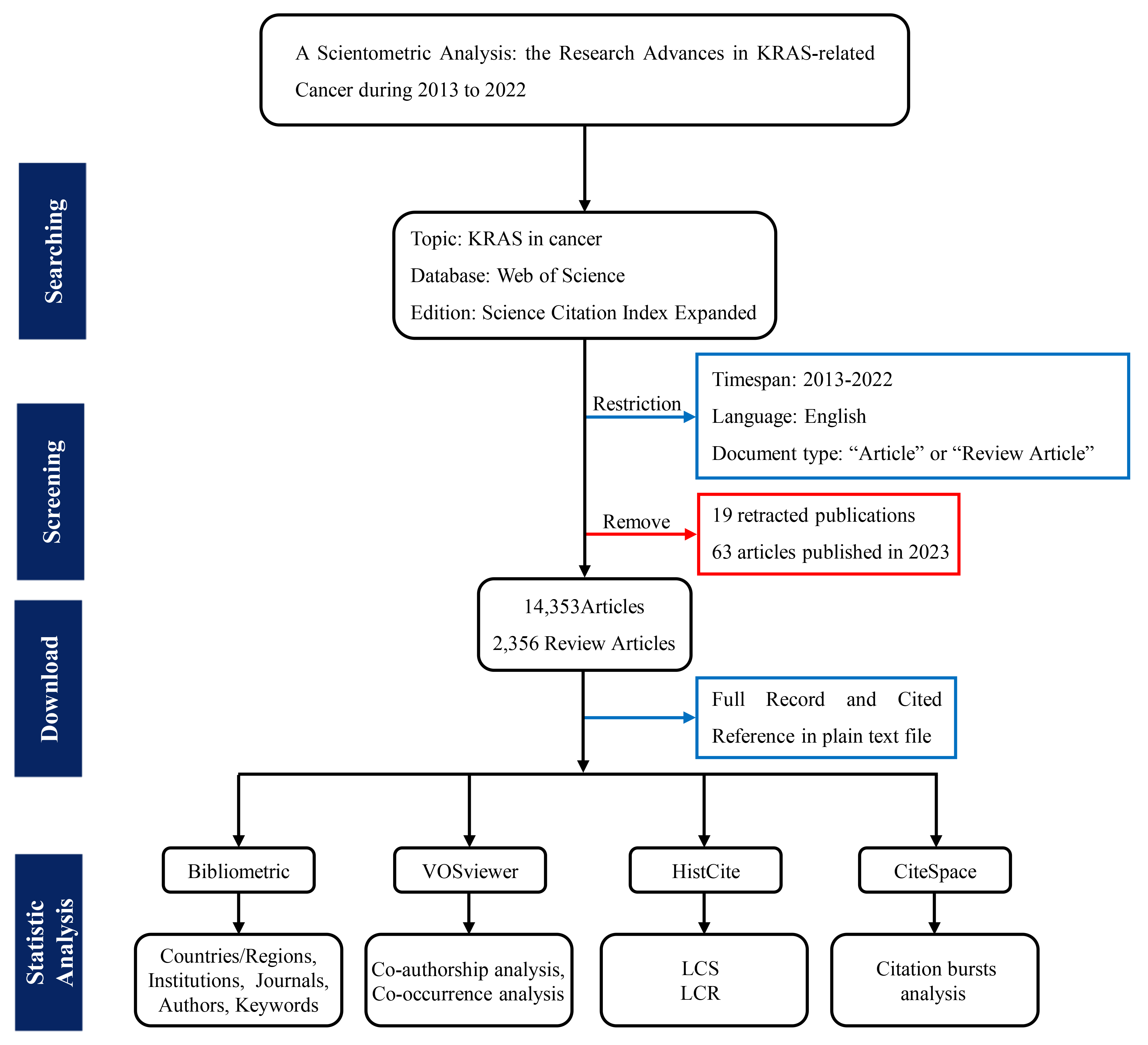
**

**Supplementary Figure 1** | Flow chart of the data collection and screening process for the statistic analysis.


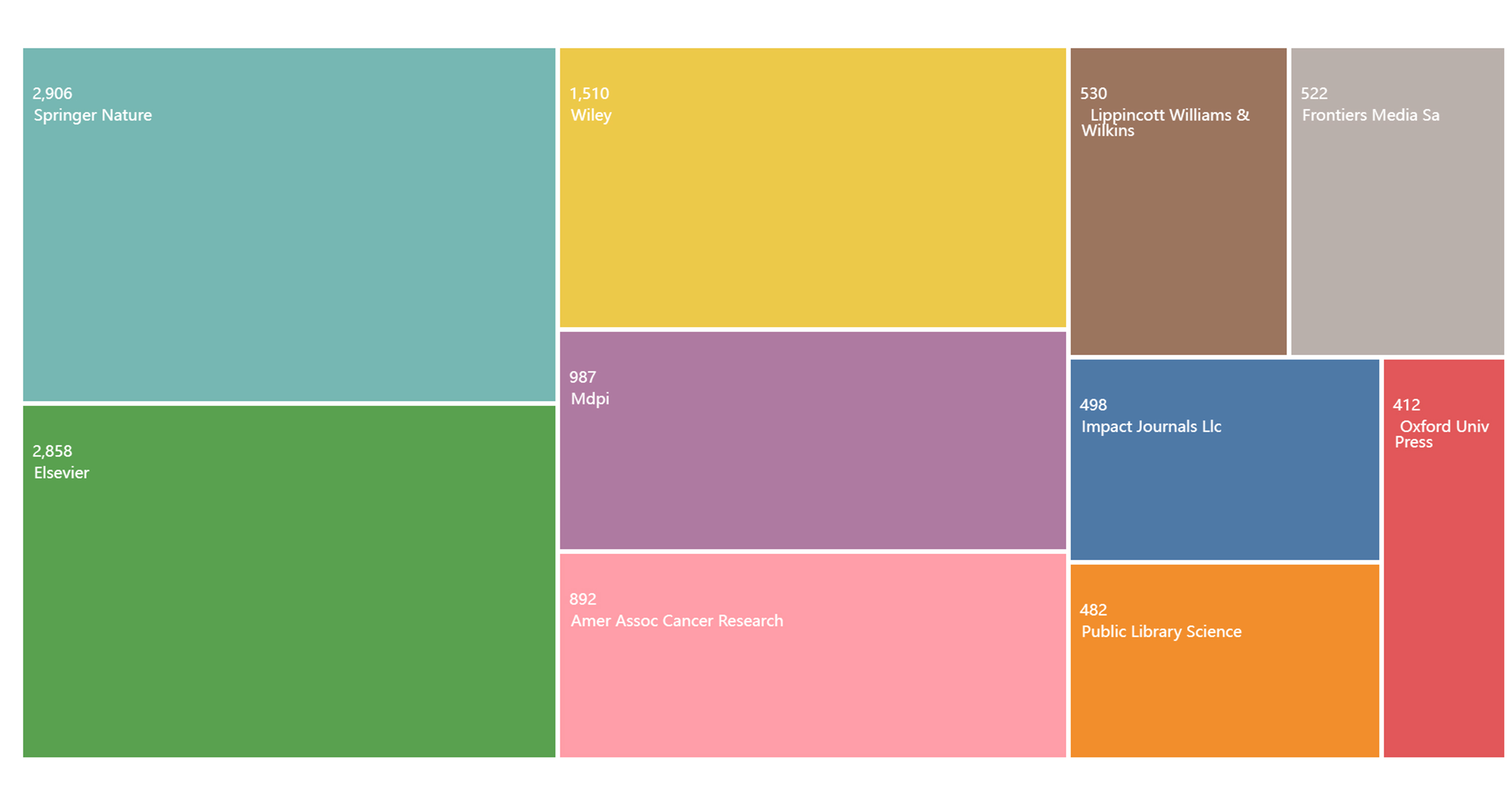


**Supplementary Figure 2** | Treemap of the top 10 most productive publishers in the research of *KRAS*-related cancer during 2013 to 2022.


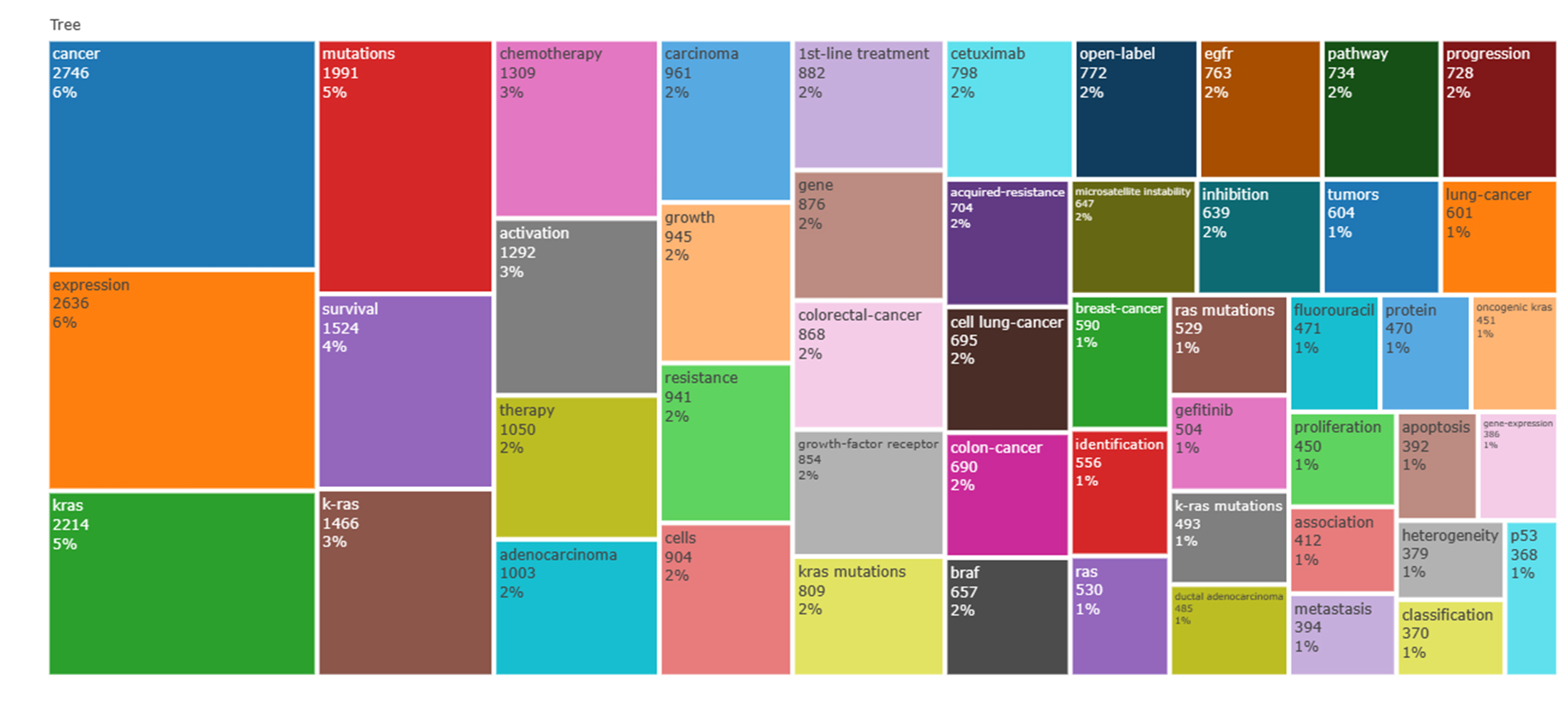


**Supplementary Figure 3** | The frequency of keywords in a tree map.
